# Differential impact of multi-focus fan beam collimation with L-mode and conventional systems on the accuracy of myocardial perfusion imaging: Quantitative evaluation using phantoms

**Published:** 2013

**Authors:** Hideo Onishi, Norikazu Matsutomo, Yoshiharu Kangai, Tatsunori Saho, Hizuru Amijima

**Affiliations:** 1 Program in Health and Welfare, Graduate on School of Comprehensive Scientific Research, Prefectural University of Hiroshima; 2 Program in Biological System Sciences, Graduate on School of Comprehensive Scientific Research, Prefectural University of Hiroshima; 3 Department of Radiology, Kurashiki Central Hospital; 4 Department of Nursing, Hyogo University of Health Sciences

**Keywords:** Multi-focus fan beam, Collimator, Image quality, Acquisition mode

## Abstract

**Objective(s):**

A novel IQ-SPECT™ method has become widely used in clinical studies. The present study compares the quality of myocardial perfusion images (MPI) acquired using the IQ-SPECT™ (IQ-mode), conventional (180° apart: C-mode) and L-mode (90° apart: L-mode) systems. We assessed spatial resolution, image reproducibility and quantifiability using various physical phantoms.

**Methods:**

SPECT images were acquired using a dual-headed gamma camera with C-mode, L-mode, and IQ-mode acquisition systems from line source, pai and cardiac phantoms containing solutions of ^99m^Tc. The line source phantom was placed in the center of the orbit and at ± 4.0, ± 8.0, ± 12.0, ± 16.0 and ± 20.0 cm off center. We examined quantifiability using the pai phantom comprising six chambers containing 0.0, 0.016, 0.03, 0.045, 0.062, and 0.074 MBq/mL of 99m-Tc and cross-calibrating the SPECT counts. Image resolution and reproducibility were quantified as myocardial wall thickness (MWT) and %uptake using polar maps.

**Results:**

The full width at half maximum (FWHM) of the IQ-mode in the center was increased by 11% as compared with C-mode, and FWHM in the periphery was increased 41% compared with FWHM at the center. Calibrated SPECT counts were essentially the same when quantified using IQ-and C-modes. IQ-SPECT images of MWT were significantly improved (P<0.001) over L-mode, and C-mode SPECT imaging with IQ-mode became increasingly inhomogeneous, both visually and quantitatively (C-mode vs. L-mode, ns; C-mode vs. IQ-mode, P<0.05).

**Conclusion:**

Myocardial perfusion images acquired by IQ-SPECT were comparable to those acquired by conventional and L-mode SPECT, but with significantly improved resolution and quality. Our results suggest that IQ-SPECT is the optimal technology for myocardial perfusion SPECT imaging.

## Introduction

Myocardial ischemia, infarcts and viability are widely assessed using myocardial perfusion imaging (MPI) using single photon emission computed tomography (SPECT). Nuclear cardiology pioneers have developed several new technologies with which to perform myocardial perfusion SPECT. Until recently, myocardial perfusion SPECT studies were extensive procedures required at least 15-20 minutes to acquire stress and rest images and generate adequate imaging statistics. Patient comfort was compromised because the arms had to be kept raised above the head during acquisition. Various manufacturers have recently introduced new dedicated hardware camera systems with optimized acquisition geometry, collimator design and associated reconstruction software to alleviate this problem.

Advances in hardware and software have reduced imaging or dose duration and improved patient comfort as well as image quality. With respect to software, Jinghan *et al* (1) reported that matched filtering improves SPECT resolution, and Hughes *et al* (2) and Onishi *et al* (3) validated image quality using a novel resolution recovery method in phantom studies. Myers *et al* (4), DePuey *et al* (5), and Ali *et al* (6) showed that these reconstruction methods clinically reduced the amount of time needed for acquisition and the radiation dose. With respect to hardware, several cameras have increased photon sensitivity achieved via customized detector geometry (7) and innovative collimation design (8, 9), focusing the field of view on the heart and using solid state detectors (10). Optimized geometry for cardiac imaging has significantly reduced or totally eliminated detector motion, with the simultaneous collection of counts from the entire heart. The IQ-SPECT™ (11) has a combined astigmatic collimator (SMARTZOOM) (12), optimized organ-of-interest centered acquisition and iterative reconstruction with CT-based attenuation correction, and it has been applied in clinical studies. Corbette *et al* (13) found in a single–center clinical trial that IQ-SPECT™ provided better quality images than conventional SPECT.

The current study quantifies the differential impact of IQ-SPECT™ (IQ-mode) conventional (180° apart: C-mode) and L-mode (90° apart: L-mode) systems in myocardial perfusion imaging, as well as the effect of spatial resolution, image reproducibility and quantifiability using various physical phantoms.

## Materials and Methods

### 

#### Study design

This study qualitatively compares the quality of myocardial perfusion images using a dual-headed camera with C-mode, L-mode, and IQ-mode systems. We evaluated image quality in terms of spatial resolution, image reproducibility and quantifiability using line source, pai, and myocardial torso phantoms, respectively.

#### Phantom study

#### Line source phantom

Since the tomographic spatial resolution of gamma cameras is often analyzed using acquisition with small line sources (capillary tubes) placed in air, we also performed capillary tube experiments. Spatial resolution was evaluated at full width at half maximum (FWHM) determined from an in-house line source phantom. Eleven capillary tubes (15 mm long and 12 mm in diameter) containing Tc-99m (20 – 25 MBq per tube) were positioned on a Styrofoam support on the camera scanning bed parallel to the axis of rotation of the camera in the center of the orbit and at ± 4.0, ± 8.0, ± 12.0, ± 16.0 and ± 20 cm off center.

#### Pai phantom

A pai phantom shaped like a pie-chart divided into six chambers was symmetrically positioned in a cylinder 160 mm in diameter and 100 mm in length. Each chamber contained 480 mL of homogeneous solutions of 0.0, 0.016, 0.03, 0.045, 0.062, and 0.074 MBq/mL ^99m^Tc. Linear regression was then analyzed between the actual and measured radioactivity activity concentrations (image quantitation).

#### Myocardial torso phantom

The conditions of capillary tube experiments do not realistically represent those of patients. Thus, we used a myocardial torso phantom to more closely model patient scans with photon attenuation and scatter in a non-uniform medium, while maintaining control over the true activity distribution. The commercially available, model RH2 elliptical cylinder torso phantom (320 mm wide and 220 mm thick) has simulated bone, lung, mediastinum, hepatic, and myocardial regions (Kyoto Kagaku Co. Ltd., Kyoto, Japan). The mediastinum, left ventricle, right ventricle and hepatic areas were filled with water. Assuming that 740 MBq of ^99m^Tc tracer was administered, the myocardial area would contain 74.2 KBq/mL. The phantom also contained a 170-mL insert modeling a healthy myocardium (without perfusion defects).

#### Image acquisition and processing

Datasets were acquired during SPECT studies using Symbia T16 SPECT/CT dual-headed gamma cameras (Siemens AG, Erlangen, Germany) equipped with a low energy high resolution (LEHR) collimator (FWHM 7.4 mm at the center) and with IQ-SPECT modification using SMARTZOOM collimators (FWHM 11.4 mm at the center). The radius of the circular orbit was 280 mm in both systems and saved to a SMARTZOOM™ system. C-mode and L-mode systems were equipped with LEHR collimators.

C-model and L-modes both comprised over 180°contoured orbits with 34 and 30 views per detector for 25 sec in 64 x 64 matrices and 4.80-mm pixels. IQ-mode images were acquired over 208° cardiocentric orbits with 17 views per detector for 15 sec per view in 128 x 128 matrices and 4.80-mm pixels. Pixel size was matched between the three acquisition modes. Datasets acquired on the Symbia T16 system were reconstructed on a Syngo MI Workplace (Siemens). The SPECT data were reconstructed using ordered subset expectation maximization (OSEM) with depth-dependent 3D resolution recovery (Flash3D™), and with ordered subset conjugates-gradient minimization (OSCGM) for SMARTZOOM collimators. Subsets and iterations were 1 and 30, respectively. Both images were attenuation-corrected based on images acquired immediately before SPECT with the integrated CT scanner. Patient-induced scatter was corrected using an energy-window-based estimate. The scatter correction was incorporated into an OSEM reconstruction algorithm. We used a Gaussian pre-filter (FWHM = 13 mm) in the Pai and myocardial torso phantom studies. A pre-filter was not used and neither scatter nor attenuation was corrected in the study of the line source phantom. The reconstruction parameters used the setting recommended by the manufactures in clinical study. Table 1 summarizes these conditions.

**Table 1:** Acquisition and reconstruction conditions.

SYSTEM	Matrix size	Collimator	Scan arc (°)	Pixel size (mm)	Number of views	Radius of rotation (mm)	Acquisition (sec/view)	Reconstruction (MLEM: It = 30)	Post Filter	Correction	
										AC	SC
C-mode.	64 × 64	LEHR	360	4.8	68	280	25	Flash3D	Gaussian*	✓	✓
L-mode	64 × 64	LEHR	180	4.8	60	280	25	Flash3D	Gaussian*	✓	✓
IQ-mode	128 × 128	SMART ZOOM	208	4.8	34	280	15	OSCGM	Gaussian*	✓	✓

* FWHM = 13 mm; AC, attenuation correction (X-CT); SC, scatter correction.

#### Evaluation

Spatial resolution was determined as the FWHM of images of the line sources in representative transaxial slices of the line source phantom after Gaussian fitting. The FWHM was calculated using an in-house analytical tool based on the method specified by the NEMA standard for calculating FWHM corrected for background. Image distortion was assessed as the aspect ratio (ASR) (14) defined as the ratio of the *radial*_FWHM_ and *tangential*_FWHM_ in the radial and tangential directions of FWHM at each position. Furthermore, C-mode acquisition datasets were reconstructed with filtered back projection used a ramp filter and non pre- and post-filters (C-mode_FBP), as compared with IQ-SPECT.

We acquired SPECT data in air using a 10-mm diameter calibration source and measured activity in C-, L-, and IQ-modes. SPECT counts were measured within a circular ROI with a radius measuring: rod radius + (FWTM of SPECT image)/2). We then computed the cross-calibration factor (CCF) as Bq/SPECT counts. Image quantifiability was validated with the linearly cross-calibrated SPECT value (Bq) vs. the radioactivity concentration (Bq) of each hot chamber in the pie-phantom for each system.

We performed myocardial image resolution to determine myocardial wall thickness (MWT) when FWHM was calculated using a profile of 6° per step generated from the short axis slices. Profiles were drawn through four slices of the short axis in the mid-ventricular region of reconstructed myocardial phantom images. Myocardial wall thickness was determined by fitting each profile to a Gaussian model. To evaluate myocardial image resolution, the MWT was defined as FWHM values of myocardial wall thickness in the torso phantom. The true MWT was calculated from the myocardial areas of short-axis images in the myocardial torso phantom using CT datasets.

Data were quantified from myocardial torso phantom SPECT images using the American Heart Association 17-segment (15) model for the left ventricle. Polar maps were normalized to 100% peak activity and the relative ratio (%) of gamma ray count uptake (%uptake) was assessed for each segment. In addition, homogeneity and mismatches were quantified in the three datasets by comparing the 17-segment values. The homogeneity, (*h (n)*) index for each segment was calculated as:


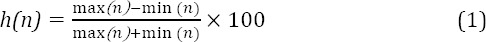


Where *n* represents the maximal and minimal values measured for each segment. The value of a completely uniform segment based on this equation is zero.

The reference image was the C-mode %uptake and the process images were L-mode and IQ-mode %uptake for each segment, respectively. A mismatch (m (s)) was calculated using equation (2).


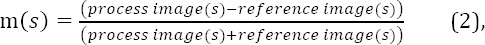


#### Statistical analysis

All data are shown as means ± standard deviation (SD) and were analyzed by ANOVA followed by the Bonferroni test. Probability values of <0.05 were considered statistically significant. All data were analyzed using SPSS 18.0 (IBM Corp., Chicago, IL, USA) software. Image quantifiability between SPECT values and radioactivity values for the pai phantom were assessed using linear regression analysis.

## Results

**Figure 1:**
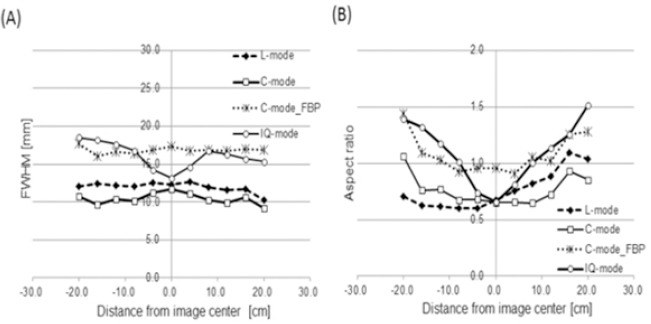
Estimated FWHM and ASR at each location of line sources using acquisition C-, L-, and IQ-modes. (A) Estimated FWHM in radial position. We added FWHM of the filtered back projection (FBP) reconstructed data (C-mode_FBP). Value of FWHM at C-mode_FBP did not depend on location and was reasonably uniform. (B) Estimated ASR tended to increase away from center. C-mode_FBP: C-mode acquisition with FBP reconstruction. ASR=tangential (FWHM) / radial (FWHM)

Figure 1 shows the FWHM (radial) and ASR of each acquisition mode in the line source phantom. The value of FWHM at different locations is shown in rough symmetry in the center of rotation. The FWHM in the C- and L-modes did not significantly vary with location. The FWHM value of IQ-mode at the central line source was increased by 11% compared with C-mode, and FWHM in the periphery was increased by 41% compared with the central FWHM. The ASR of IQ-mode was 0.6-1.5. No acquisition mode of ASR was 1.0 in the center, and those of ASR were increased more in the periphery.

**Figure 2:**
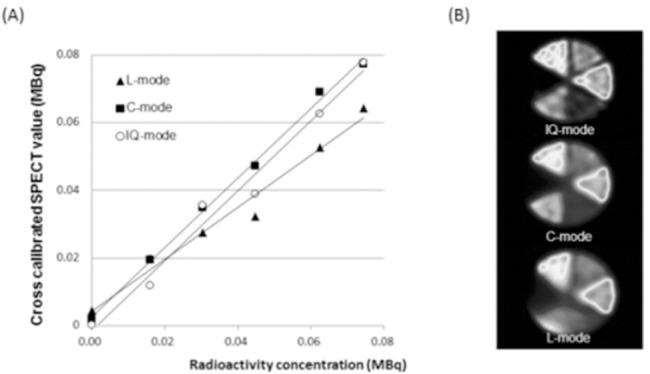
Linear regression analysis and pai phantom SPECT images. (A) Linear regression analysis shows relationship between relative SPECT counts and relative radioactivity concentrations for C-, L- and IQ-modes. (B) Pai phantom SPECT images. Top, IQ-mode; middle, C-mode; bottom, L-mode.

Figure 2 shows the correlation between cross-calibrated SEPCT values and radioactivity concentrations as a function of acquisition mode in six chambers of the pai phantom containing 99m-Tc. The results of linear regression analysis between SPECT values and the radioactivity concentrations were as follows: C-mode, Y = 1.02X + 0.002 (R^2^= 0.99); L-mode, Y = 0.76X+ 0.004 (R^2^ = 0.97) and IQ-mode; Y = 1.03X – 0.001 (R^2^ = 0.98). Compared with the results obtained from L-mode, the gradient of the regression function for C- and IQ-modes was about 1.0. The y-intercept value of IQ-mode was negative.

**Figure 3:**
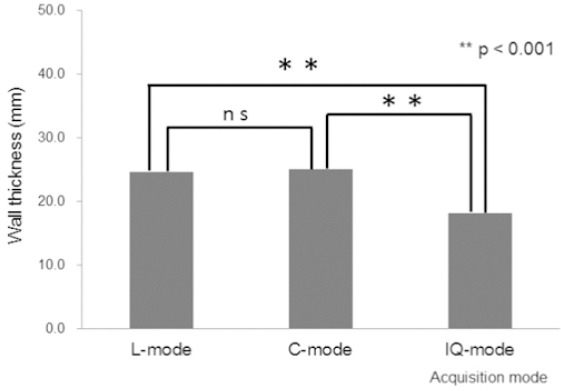
Correlations among C-, L- and IQ-modes.Myocardial wall thickness was lower with IQ-mode than C-mode and L-mode at FWHM of phantom. NS, not significant.

The true MWT measured at 20.2 ± 1.5 mm from the myocardial torso phantom was significantly higher in L- and C-, than in IQ-mode (24.6±1.0 vs. 18.2±1.2, *P*<0.001 and 25.2±1.1 vs. 18.2±1.2, *P*<0.001), respectively. Conventional and L-modes did not significantly differ (*P*=0.16) (Figure 3). The MWT in the torso phantom was significantly improved by IQ-mode, as compared with L- and C-modes.

**Figure 4:**
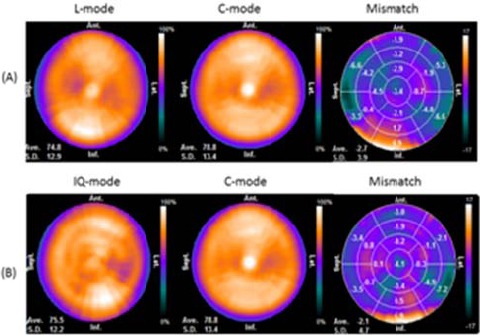
Polar map of myocardial torso phantom using 17-segment model.

Figure 4 shows three sets of polar maps (%uptake, mismatch) for C-, L-, and IQ-modes with the 17-segment model and the myocardial torso phantom. Table 2 shows the %uptake, mismatch and homogeneity of the three datasets. The maximal difference in %uptake among the three modes was 13% in the quantitative 17-segment model. L- and IQ-modes both affected the mismatch more than C-mode. In particular, basal anteroseptal, basal inferolateral, mid anteroseptal, and mid-inferolateral mismatches were significantly larger in the 17-segment model. The effects of the three modes on homogeneity significantly differed in the 17-segment model. The effect of C-mode on homogeneity in the basal, mid, and apical regions was significantly larger than that of IQ-mode (C-mode vs. L-mode: *P*=0.070, 0.061 and 0.058, respectively; C-mode vs. IQ-mode: *P*<0.05 for all three regions).

**Table 2:** Findings of uptake, mismatch and homogeneity in 17 segments using myocardial torso phantom.

Segment	Uptake (%)			Mismatch		Homogeneity (%)		
	C	L	IQ	L	IQ	C	L	IQ
1	74.7	71.7	69.8	-1.9	-3.0	32.7	34.1	36.9
2	60.3	52.8	56.5	-6.6	-3.4	39.5	41.1	44.3
3	60.7	57.1	55.5	-3.3	-3.5	36.8	41.5	47.3
4	62.7	70.9	70.3	6.9	6.9	36.9	41.3	42.6
5	65.2	57.3	56.9	-6.6	-7.2	37.3	40.7	45.6
6	61.3	55.4	58.8	-5.3	-2.1	38.4	40.7	42.1
7	90.6	85	87.2	-3.2	-1.9	5.8	6.6	8.7
8	80.7	74.2	82.0	-4.2	0.8	5.2	7.9	9.0
9	79.7	79.3	78.7	-0.4	-0.7	7.4	8.9	17.0
10	87.9	91.1	89.2	1.7	0.5	5.1	10.2	16.7
11	81.1	74.7	74.1	-4.0	-4.9	8.2	10.4	23.1
12	78.9	75.8	77.2	-1.9	-1.1	10.4	12.0	15.6
13	85.5	80.8	85.0	-2.9	-0.2	8.3	10.7	12.5
14	76.8	70.3	76.8	-4.5	0.1	8.1	11.0	15.5
15	85.0	81.6	79.5	-2.1	-3.4	11.2	13.3	14.7
16	73.1	72.0	72.7	-0.7	-0.3	10.1	12.3	16.4
17	88.0	82.1	80.7	-3.4	-4.1	13.59	17.92	18.34

C, Conventional mode; L, L-mode; IQ, IQ-mode; Seg, segment.

The segment is 1: basal anterior, 2: basal anteroseptal, 3: basal inferoseptal, 4: basal inferior, 5: basal inferolateral, 6: basal anterolateral, 7: mid anterior, 8: mid anteroseptal, 9: mid inferoseptal, 10: mid inferior, 11: mid inferolateral, 12: mid anterolateral, 13: apical anterior, 14: apical septal, 15: apical inferior, 16: apical lateral, 17: apex

## Discussion

The rapid acquisition of myocardial perfusion images can reduce the burden on patients and clinical studies have shown that the IQ-SPECT system reduces the amount of time needed to acquire myocardial perfusion SPECT images. The present assessment of image quality in C-, L-, and IQ- acquisition modes using phantoms showed that that IQ-mode can improve image resolution and quantifiability.

Image resolution in the center of the field of view was similar among the three modes, but the resolution in IQ-mode was degraded at the periphery region compared with the other modes (Figure 1A). Paramithas *et al* (16) and Imbert *et al* (17) reported that FWHM between 14 and 18 mm confers a slight improvement towards the collimator face. Our results roughly coincided with theirs when FWHM was centrally located. The differences between the central and periphery were larger for IQ-mode than for C- and L-modes; mainly because the Smartzoom focused collimator produced distortions in the absence of focused targets. These notions were supported by the findings of the ASR (Figure 1B) which at the periphery increased 1.5 fold compared with the center, and images were distorted at the periphery in the absence of focused targets. The center of the suggested ASR was not 1.0, possibly due to the low iteration updates obtained using the conditions recommended by the manufacturer for the clinical study. Imbert *et al* indicated a difference between radial and tangential FWHM (17). Our ASR findings agreed with those described by Onishi *et al* (3), who noted that SI (product of subset and iteration) >100 updates are needed for ASR to be 1.0. Resolution recovery clearly improved resolution more than FBP.

Image resolution of MWT was significantly better in IQ-mode than in C- and L-modes (Figure 3). Resolution recovery correction in C- and L-modes did not significantly improve resolution. We previously reported that resolution recovery is limited by the radii of rotation (<200 mm), and that the resolution recovery ratio decreases as the radii of rotation increase (3). On the other hand, IQ-mode slightly underestimates MWT, but was closer to the true MWT value.

We evaluated the accuracy of the three acquisition modes for quantitative imaging using a cross-calibration technique (18) (Figure 2). The gradients of the regression function for C-, L-, and IQ-modes were 1.02, 0.76, and 1.03, respectively. The accuracy of the y-intercept values was within 5% of the maximum activity of the chambers in the pai phantom. Image degradation was obvious in L-mode (Figure 3b). The accuracy of acquisition modes for quantitative imaging was the same for the C- and IQ-modes. In contrast, L-mode underestimated the SPECT count by 20% according to the gradient of the regression function.

This phantom study showed that L-mode had a considerable and negative impact on image homogeneity and the accuracy of image quantitation. Imaging the myocardial torso phantom using L-mode significantly increased %uptake and basal anteroseptal, basal inferolateral, mid anteroseptal, and mid inferolateral mismatches compared with C-mode because the myocardial region was positioned off center in the phantom (Figure 4). Liu *et al* (19) revealed significant erroneous homogeneity and artifacts when the target object is off the center of a 180° orbit. In contrast, the %uptake and homogeneity did not change with C-mode in the segments (Table 2), because the acquisition orbit was 360° and SPECT images were reconstructed from 360° projection datasets. The IQ-mode sets the rotational center at the cardiac region and thus improved %uptake and mismatch compared with L-mode. A slight mid-inferolateral mismatch (4.9%) that occurred in IQ-mode might have been due to the acquisition angle being limited to a 208° arc.

The IQ-mode equipped with Smartzoom system increase sensitivity and resolution (16) and decreased homogeneity more significantly (*p* <0.05) than C-mode.

The performance (accuracy and homogeneity) of IQ-mode is the same as C-mode and is thus useful for acquiring MPI. In addition, performance is maintained during a short acquisition period (16). However, although the myocardial torso phantom is representative of a normal human chest, upward creep of the heart provoked reconstruction errors and created artifacts. Other artifacts such as those arising from motion generated by patients or the heart during acquisition for CT attenuation correction (20) are unpredictable.

We consider that IQ-mode is useful for myocardial perfusion imaging despite decreasing projection datasets and orbit issues.

## Conclusion

The quality of myocardial perfusion imaging was comparable among IQ-mode, C-mode and L-mode SPECT, with significantly improved image resolution and quality. This study provides new and important information about image resolution and quality using a multi-focus fan beam collimator system (IQ-SPECT), which might be the most effective technology for myocardial perfusion SPECT imaging.
